# Embedded Electronic Sensor for Monitoring of Breathing Activity, Fitting and Filter Clogging in Reusable Industrial Respirators

**DOI:** 10.3390/bios12110991

**Published:** 2022-11-08

**Authors:** Pablo Aqueveque, Macarena Díaz, Britam Gomez, Rodrigo Osorio, Francisco Pastene, Luciano Radrigan, Anibal Morales

**Affiliations:** 1 Department of Electrical Engineering, Faculty of Engineering, Universidad de Concepción, 219 Edmundo Larenas St., Concepción 4030000, Chile; 2 Department of Multidisciplinary Engineering, Faculty of Engineering, Universidad de Santiago de Chile, Libertador Bernardo O’Higgins Av., Santiago 9170022, Chile; 3 Department of Electrical Engineering, Universidad Católica de la Santísima Concepción, Concepción 4090541, Chile

**Keywords:** breathing monitoring, industrial monitoring, occupational safety, embedded monitoring sensor

## Abstract

Millions of workers are required to wear reusable respirators in several industries worldwide. Reusable respirators include filters that protect workers against harmful dust, smoke, gases, and vapors. These hazards may cause cancer, lung impairment, and diseases. Respiratory protection is prone to failure or misuse, such as wearing respirators with filters out of service life and employees wearing respirators loosely. Currently, there are no commercial systems capable of reliably alerting of misuse of respiratory protective equipment during the workday shifts or provide early information about dangerous clogging levels of filters. This paper proposes a low energy and non-obtrusive functional building block with embedded electronics that enable breathing monitoring inside an industrial reusable respirator. The embedded electronic device collects multidimensional data from an integrated pressure, temperature, and relative humidity sensor inside a reusable industrial respirator in real time and sends it wirelessly to an external platform for further processing. Here, the calculation of instantaneous breathing rate and estimation of the filter’s respirator fitting and clogging level is performed. The device was tested with ten healthy subjects in laboratory trials. The subjects were asked to wear industrial reusable respirator with the embedded electronic device attached inside. The signals measured with the system were compared with airflow signals measured with calibrated transducers for validation purposes. The correlation between the estimated breathing rates using pressure, temperature, and relative humidity with the reference signal (airflow) is 0.987, 0.988 and 0.989 respectively, showing that instantaneous breathing rate can be calculated accurately using the information from the embedded device. Moreover, respirator fitting (well-fitted or loose condition) and filter’s clogging levels (≤60%, 80% and 100% clogging) also can be estimated using features extracted from absolute pressure measurements combined to statistical analysis ANOVA models. These experimental outputs represent promising results for further development of data-driven prediction models using machine learning techniques to determine filters end-of-service life. Furthermore, the proposed system would collect relevant data for real-time monitoring of workers’ breathing conditions and respirator usage, helping to improve occupational safety and health in the workplace.

## 1. Introduction

Industrial workers get constantly exposed to different agents in their workplaces, such as dust and vapors, which act as irritants, carcinogens, or immunological agents [1,2]. The National Institute for Occupational Safety and Health (NIOSH) from United States of America (USA) declares that the prevention of exposure to dangerous particles using Personal Protective Equipment (PPE) and the monitoring of environmental conditions are the primary methods of controlling respiratory diseases on workers. However, workers may still be affected by those dangerous particles due to extended time exposure, defective PPE, and previous worker’s health condition [3]. Respiratory conditions due to toxic agents have been reported as one of the three leading causes of occupational illnesses in the United States, leading to 508.4 thousand cases within all industries during 2020 [4]. According to the U.S. Bureau of Labor Statistics, in 2020, 672 workers died due to exposure to harmful substances or environments [5].

Workers’ health monitoring is crucial for preventing respiratory illnesses. Medical respiratory techniques used for this purpose are radiography, spirometry, questionnaires, and bio-markers [3]. However, those methods do not perform continuous measurements in actual conditions since they require workers to leave their tasks to perform the corresponding measurements. Since PPE are critical for industrial workers, there is commercially available equipment that quantifies the respirator fitting and detects air leaks, such as the PortaCount^®^ Respirator Fit Tester (TSI Incorporated, Shoreview, MN, USA) [6], which evaluates the seal between the face and the respirator. The equipment performs the test outside the work area and before the workday, so if the respirator loses its fit or is damaged, it is not possible to monitor the exposure to potentially harmful environments during the work-shift [7].

To assess the issue concerning real-time and on-site breathing monitoring, wearable systems have been developed for clinical applications mostly. Researchers have developed new devices that can measure breathing variables in a non-invasive way. Different sensors have been used to interpret changes in the body (or a field near the body) to track breathing. The chest movements during breathing produce changes of capacitance in the thorax cycle that can be measured using impedance measurement systems [8,9,10]. Other devices estimate the mechanical oscillations of the thorax during breathing using inertial sensors, strain gauges, inductance plethysmography, or flexible resistive bands [11,12,13,14,15]. In general, the systems are accurate and can perform breathing monitoring correctly. However, these devices may not be suitable for industrial environments as the electrodes positioned over the worker’s body may change their position during the activities. Moreover, their clothes cannot be intervened for adding strain gauges, and adhesive stripes may cause discomfort while working. Other solutions measure temperature near the respiratory tract where expired air has a greater temperature than inspired, suitable for wearables and non-invasive embedded systems [16,17].

Industrial respirators and face masks are a key implement to develop wearable breathing monitoring systems. Xu et al. [18] developed a small sensor to be used in the exhalation valve of a KN95 mask. The sensor measures the periodic airflow temperature variations caused by exhaled hot air and inhaled cool air in respiratory cycles. The work focuses on the sensor and algorithm development for assessing the user’s breathing frequency. Although this sensor may be suitable to use in the industrial environment, it does not allow for assessing the respirator fitting and the filter clogging level. Lo Presti et al. [19] developed a fiber Bragg grating-based sensor based on nasal airflow changes detection from humidity signals. Although the sensor offers a comfortable and minimally invasive way of monitoring breathing frequency, it is located under the nostrils and is attached to the user’s ears, which is not suitable for workers during their shifts. In [20], a biomask with integrated sensors is presented as a device compatible with electroencephalography, electromyography, electrooculography, and electrocardiogram for patients in clinical environments. The study [21] shows a patent for a sensorized mask for CPAP machines that measures physiological variables. These two proposals show innovative designs of sensorized respirators with clinical applications, but none can be applied to industrial environments. A patent [22] shows a wearable mask fit monitor that measures particle concentrations inside and outside the respirator. This device does not indicate the breathing rate measurement during work-shifts.

The main breathing monitoring techniques in clinical practice are pulse oximetry, pressure/volume curves, electric impedance tomography, and diaphragmatic electromyography, which have shown high reliability in assessing the patient respiratory system [23]. However, none of these measurement techniques and devices have been applied to workers in industrial sites due to these devices require special conditions in a controlled environment.

Considering that in some industries, such as the mining industry, the work shifts can last up to 12 hours [24,25], it is essential to implement continuous breathing monitoring and environmental monitoring techniques to complement PPE [3,6,26]. The continuous monitoring during several hours summed to the strict regulations on the use of wireless networks represents a challenge for implementing new monitoring devices within the workplace, due to the presence of several networks with low bandwidth and high saturation [27,28].

This paper proposes a novel wearable breathing electronic sensor inside a half-face-piece respirator. This sensor inside a PPE commonly used in industrial environments [5] measures the pressure (P), temperature (T), and relative humidity (rH) to continuously monitor workers’ breathing and environmental conditions in their workplace. From now on the paper we use P for pressure signal, T for temperature signal and rH for relative humidity signal. The objective is to evaluate the level of protection of their PPE, discriminating between fitting conditions and differentiates levels of filter clogging.

## 2. Material and Methods

### 2.1. Electronic Design

**Figure 1:**
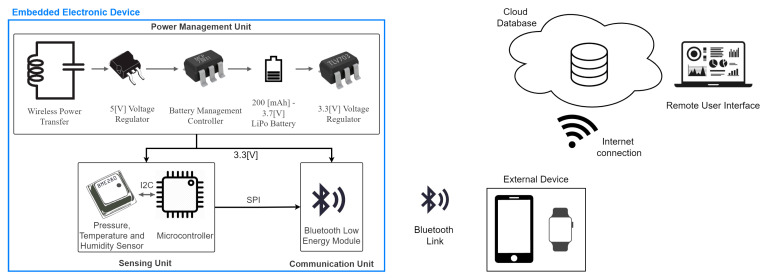
Hardware diagram of the proposed system using the embedded electronic device.

The proposed embedded electronic device uses integrated pressure (P), temperature (T), and relative Humidity (rH) sensors to measure breathing. These sensors are controlled by a low-power MicroController Unit (MCU), which processes and sends the signals to an external platform through a Bluetooth Low-Energy (BLE) module. The device is powered by a 3.7 V-100 mAh LiPo battery (Shenzhen EPT Battery CO. LTD., Shenzhen, China), which is charged through a wireless power transfer system (WPT). The general diagram of the proposed embedded electronic device is shown in Figure 1. This device is designed to be connected to a smartphone or smartwatch of a worker using a BLE link. Thus, the device will alert the worker in case of any breathing or PPE problem (respirator fitting or filter clogging). Furthermore, through an internet connection, the smartphone or smartwatch sends the information to a cloud database where the worker supervisor can monitor multiple worker’s breathing and respirator condition.

#### 2.1.1. Sensing Unit

An STM32L422CBT6 (STMicroelectronics, Geneva, Switzerland) MCU was used due to its processing capabilities and communication interfaces, suitable for low-power applications [29].

To measure the P, T, and rH, the BME280 (Bosch Sensortec, Reutlingen, Germany) was selected. This sensor allows measuring pressure (P) between 30–110 kPa with a 20-bit resolution, temperature (T) between −40–85 °C with a 20-bit resolution, and relative humidity (rH) between 0–100% with a resolution of 0.008% [30].

The MCU configures the sensor to acquire the signals (P, T, rH) at 10 Hz and manage the wireless communication on a server-client type link, sending the collected data to an external device in real-time.

The firmware task works as follows:

- The MCU initialize the system and configure the BLE module for advertising mode, waiting for a connection attempt.
- The MCU uses a timer interruption, configured at 10 Hz for sampling P, T, and rH signals.
- In real-time, if there is a BLE connection, the MCU send the samples measured and the device battery level to an external device (P.C. or Smartphone).
- In case of any failure, the system continues working except in a power failure where the embedded electronic device is turned off.

#### 2.1.2. Wireless Communication Unit

For the BLE protocol, the embedded electronic device uses the BlueNRG-M2SA module (STMicroelectronics, Geneva, Switzerland). This module implements the Bluetooth 5.0 stack, allowing the maximum efficiency between the distance of connection and power consumption [31]. The BLE module is configured minimizing the antenna power achieving a maximum connection distance of 3 m (−2 dB of TX power), enough to achieve robust communication between the worker respirator and its smartphone. However, as the BlueNRG-M2SA module is a class 2 Bluetooth device, it can achieve a wireless communication distance of up to 10 m when the TX power is +2 dB. In the case where it is required a longer distance, the BLE module of the embedded electronic device can be replaced with a class 1 Bluetooth device, which allows a wireless communication distance up to 100 m.

#### 2.1.3. Power Supply Management Unit

The proposed embedded electronic device uses a 3.7 V-100 mAh LiPo battery of 3 mm × 30 mm × 25 mm. The WPT uses an inductive link in the series-parallel topology [32,33]. The inductive link’s operational frequency is 1 MHz. The receiver coil is fabricated in a rigid PCB and has a radius of 7.75 mm, an inductance of 0.892 µH, and a resistance of 1.145 Ω. After the coil, an L7805ABD2T-TR (STMicroelectronics, Geneva, Switzerland) power management device is used to energize with 5V an MCP7383 (Microchip, Chandler, AZ, USA) LiPo battery charger. The TLV703 (Texas Instruments, Dallas, TX, USA) 3.3 V voltage regulator is used to supply all the resting devices.

#### 2.1.4. Electronic Sensor Implementation

The electronic design was implemented in a 1/2 [oz] double layer rigid copper PCB of 0.8 mm thickness. An LPKF ProLaser S Laser structuring machine (LPKF Laser & Electronics, Garbsen, Germany) was used to build the PCBs. The electronic device was separated into three PCBs to fill the space optimally. One PCB contains the BME280 chip sensor only because this sensor has to be exposed to the air and free of epoxy resin. Other PCB contains the Power Management Unit, including the wireless power transfer coil, which must be inside the resin block’s border. Finally, the third PCB contains the microcontroller and the Communication unit.

The power consumption of the embedded electronic device is about 9.9 mW during active BLE communication, which gives an autonomy of 30 [h] working continuously when the device is powered by 100 mAh LiPo battery.

### 2.2. Encapsulation Process

The electronic system was embedded in a bio-compatible epoxy resin block of 27 mm × 37 mm × 15 mm in order to allow its location inside an industrial respirator. The equipment incorporated in the standardized manufacturing process consists of a glass vacuum chamber, a vacuum pump and vacuum lines (hoses). The encapsulation process is to homologate the IP66 (Ingress Protection N°66) norm required for its use in industrial environment [34,35]. After 6 hours of curing, the completely encapsulated measurement system is obtained, without the presence of bubbles inside the electronics block.

**Figure 2:**
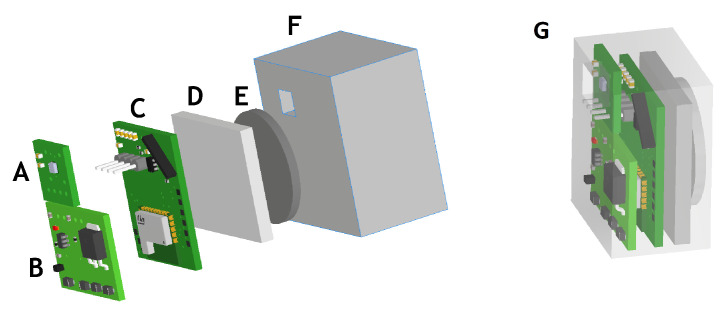
3D model of the mechanical implementation for the device. (**A**) BME280 PCB (**B**) Power management unit PCB (**C**) Microcontroller and wireless communication unit PCB. (**D**) LiPo battery. (**E**) Ferromagnetic metal part. (**F**) Epoxy resin. (**G**) Embedded electronic device.

**Figure 3:**
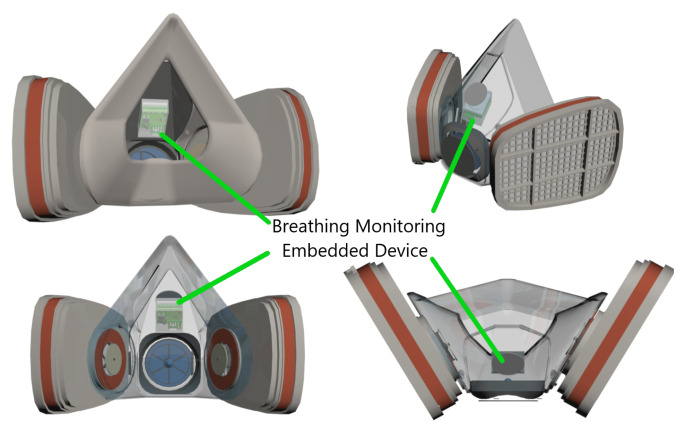
Isometric views of the reusable industrial respirator with the embedded electronic device magnetically attached using an external magnet.

**Figure 4:**
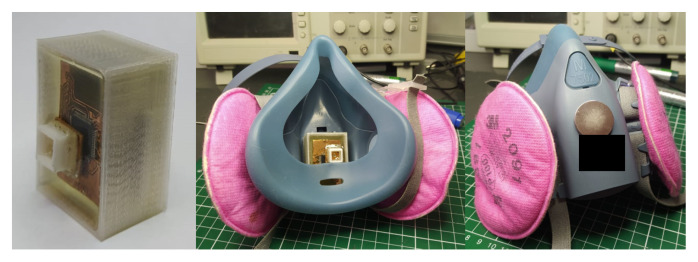
Implemented breathing monitoring embedded electronic device (**left**) inside a reusable respirator (**center**), where the positioning of the system is through an externally located magnet (**right**). The first image shows the embedded electronic device with epoxy resin.

Figure 2 shows a 3D model of the embedded electronic device, while Figure 3 illustrates the device’s location inside a reusable respirator. The implemented breathing monitoring embedded electronic device located inside an industrial respirator is shown in Figure 4.

### 2.3. Experimental Validation

#### 2.3.1. Participants

10 healthy adults (ages 24 to 30 years old; 6 males, 4 females) with no record of chronic pulmonary disease and non-smokers volunteered to participate in this study.

Informed consent was signed by the participants. The study was conducted according to the guidelines of the Declaration of Helsinki, and approved by Ethics Committee of the research and development from Universidad de Concepción (CEBB 838-2020).

#### 2.3.2. Materials

**Figure 5:**
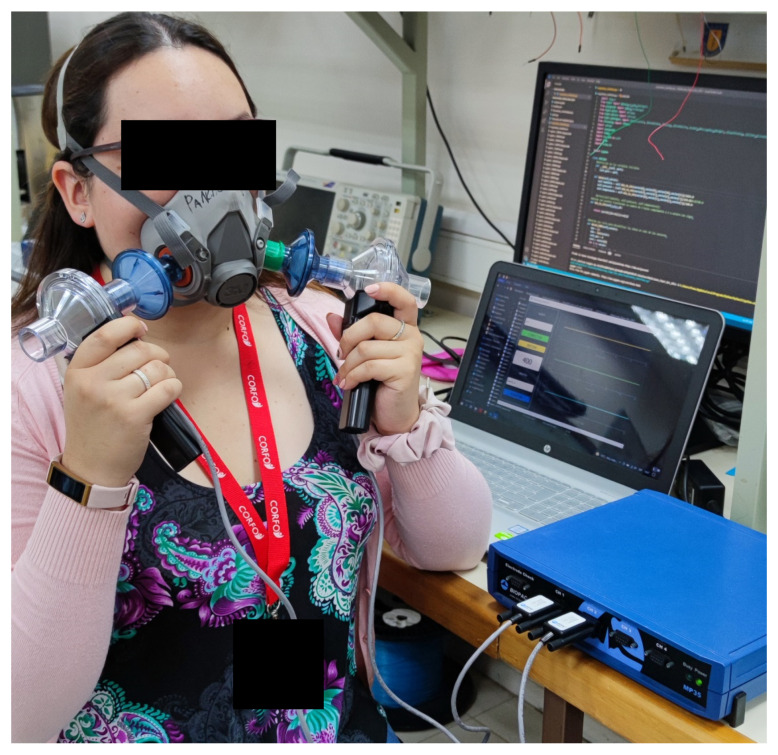
Experimental setup for signals acquisition on a participant.

Signals were acquired simultaneously by the embedded electronic device and a TSD117B airflow, lung volume, and expired gas transducer (BIOPAC Systems Inc., Goleta, CA, USA) connected to an Biopac MP35 signals acquisition system (BIOPAC Systems Inc., Goleta, CA, USA). The airflow transducer was adapted to the reusable industrial respirator to acquire airflow (AF) as a reference signal (see Figure 5).

#### 2.3.3. Data Recording Protocol

**Figure 6:**
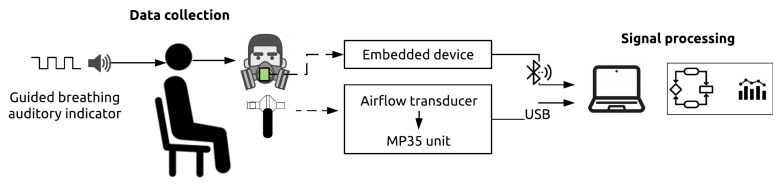
Schematic diagram of experimental setup for data collection process.

To validate the proposed embedded electronic device, a controlled breathing rate protocol was carried out considering the normal spontaneous breathing rate in adults (12-20 breaths per minute (BPM)) [36]. The embedded electronic device performance was evaluated in terms of breathing rate measurements, respirator’s fitting condition discrimination and filter clogging categorization. At the beginning of each protocol, the subjects were asked to take a deep breath to synchronize the signals. They were asked to inhale and exhale through their nose during the recording process while they remained seated in a relaxed position. A photo and a diagram of the experimental setup are shown in Figure 5 and Figure 6, respectively.

**Figure 7:**
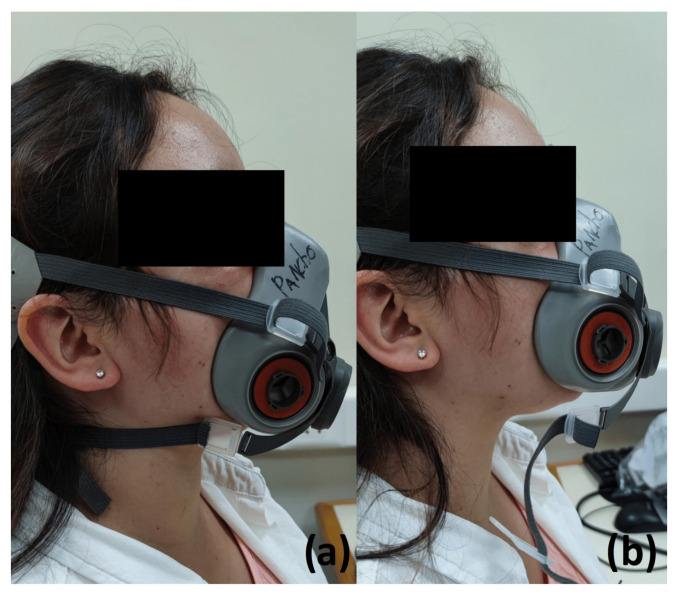
Volunteer with the reusable respirator in fitted (**a**) and loose (**b**) position.

**Figure 8:**
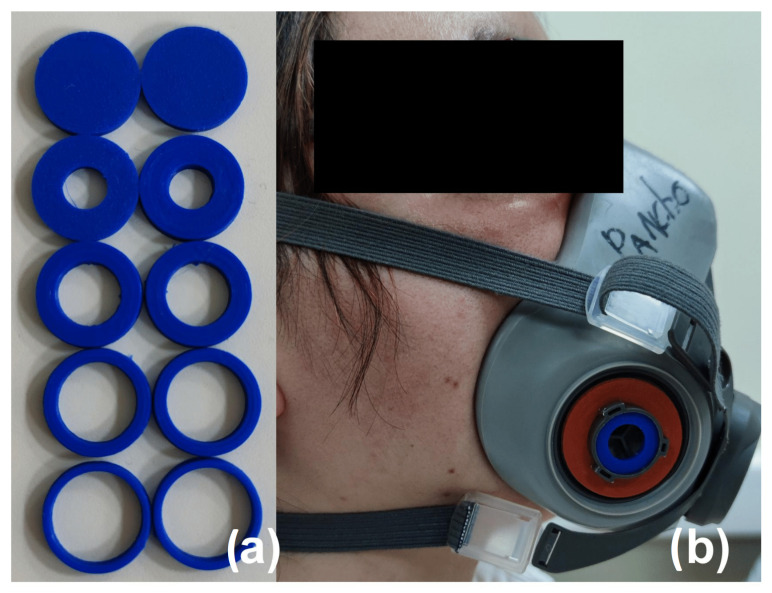
(**a**) Plastic pieces for simulating the filter clogging and (**b**) a volunteer using the respirator with the filter clogged.

1. Breathing rate evaluation
  The participants remained seated in a relaxed position and synchronized their breathing with an auditory indicator, which marked the beginning and the end of inspiration and expiration. Rate values of 10, 13, 16, 19, and 22 BPM were used. The recording lasted for 5 minutes for each breathing rate.
2. Fitting condition evaluation
  The participants remained seated in a relaxed position. The subjects were asked to breathe using a fitted respirator, named as Initial Fitted condition. After 5 min of recording, the respirator stripes were loosened (See Figure 7). Ten minutes after, the respirator stripes were tightened again, named as Final Fitted condition. The spontaneous breathing of the participant was acquired during 15 minutes.
3. Filter clogging evaluation
  The participants remained seated in a relaxed position. This protocol was applied six times per subject. Each time, a 3D printed PLA piece was put in the filter fixations, in the external part of the respirator. Each plastic piece represents a level of clogging (0, 20, 40, 60, 80, and 100%). Figure 8 shows the plastic pieces and an example of a plastic piece located in the respirator. The spontaneous breathing of the participant was acquired during 5 minutes for each clogging level.

### 2.4. Data Analysis

**Figure 9:**
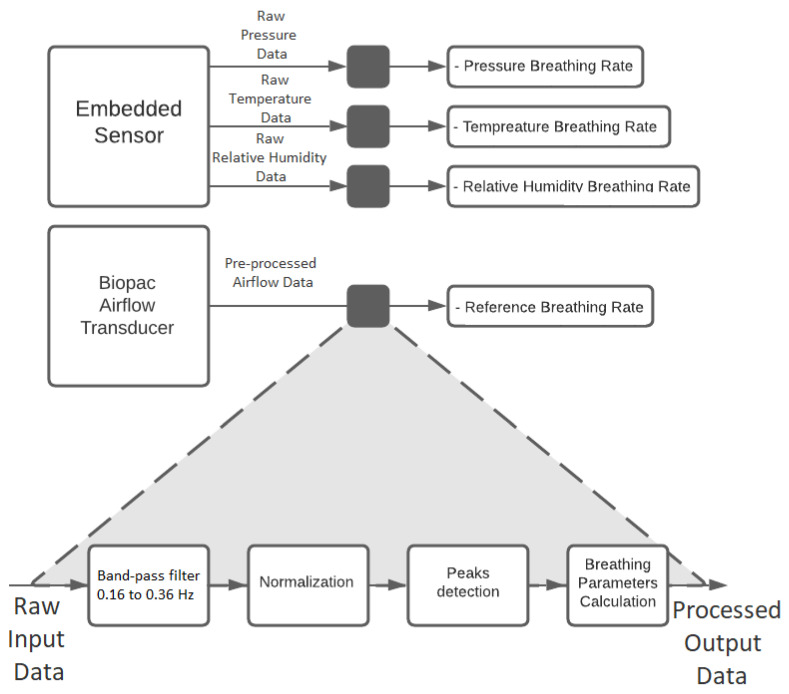
Signal processing flowchart.

**Figure 10:**
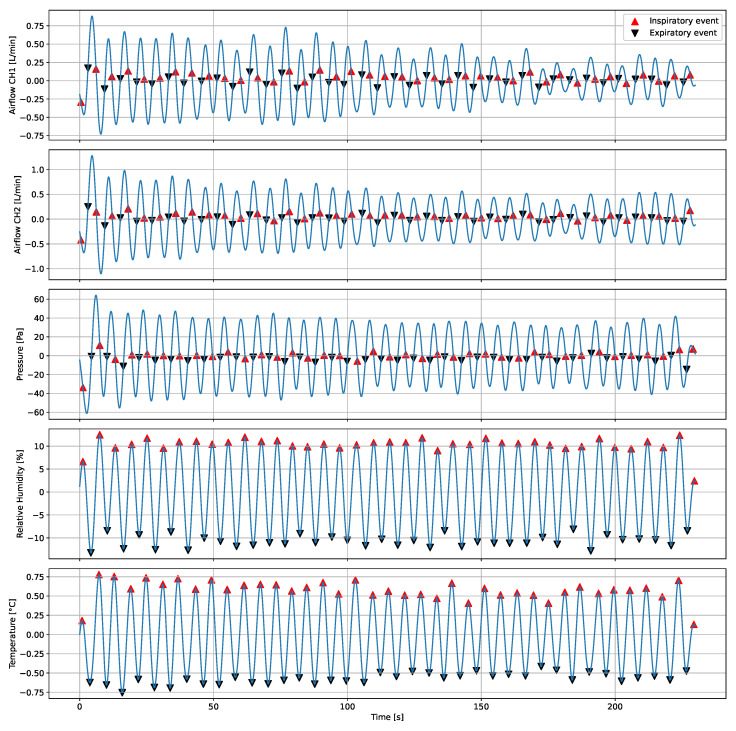
Inspiratory and expiratory events detection for one subject.

1. Breathing rate evaluation.
  Firstly, the P, T, and rH signals were filtered by a zero-phase 4th order band-pass Butterworth filter from 0.16 Hz to 0.36 Hz. The variation pattern of the sensors while recording breathing differs between them. The beggining of an inspiration (inspiratory event) for P and A.F. is given by the falling inflection point of a cycle, whereas the start of an expiration (expiratory event), by the rising inflection point. On the other hand, for T and rH the inspiratory and expiratory event correspond to the local maxima and minima of a cycle, respectively. Therefore, two algorithms were implemented to determine the inspiratory and expiratory events. A peak-detection-based algorithm was used [37] to identify the inspiratory and expiratory events on the AF and P signals’ first derivative and on the T and rH signals. Then, instantaneous breathing rate ($I_{BPM}$) was obtained calculating the inverse of the difference between the time of occurrence of local maxima ($tmax_{i}$) in seconds multiplied by 60 (see Equation (1)).
  (1) $$I_{BPM}=\frac{60}{\left(tmax_{i}-tmax_{i-1}\right)}$$
  where (*i*) corresponds to the number of the detected local maxima.
  The flowchart of the signal processing is shown in Figure 9. Figure 10 shows the A.F., P, T and rH signals and the inspiratory and expiratory events detected for one subject.
2. Fitting condition evaluation.
  Data were segmented for three conditions: (A) Initial Fitted condition, (B) Loose condition, and (C) Final Fitted condition. To assess the separability between the three conditions, a two-sided paired t-test was performed (α = 0.05) after checking normal distributions with the Shapiro–Wilk test. The T-test was applied three times. First, to assess if the initial fitted condition can be discriminated from the loose condition. Second, to assess if the loose condition can be discriminated from the final fitted condition. Third, to assess if the data distribution is the same (or similar) during the initial and final fitted conditions. For reliability and repetitivity of the fitting condition evaluation, it is expected to obtain the same data distribution during the initial fitted condition and the final fitted condition.
3. Filter clogging evaluation
  Data were grouped by clogging level: 0, 20, 40, 60, 80, and 100%. To evaluate the effect of clogging level on the peak-to-peaks values of the different signals, multiple comparisons were performed with one-way Analysis of Variance (ANOVA) followed by a post hoc comparison (α = 0.05) using Tukey’s method.

## 3. Experimental Results Analysis

### 3.1. Breathing Rate Evaluation

**Figure 11:**
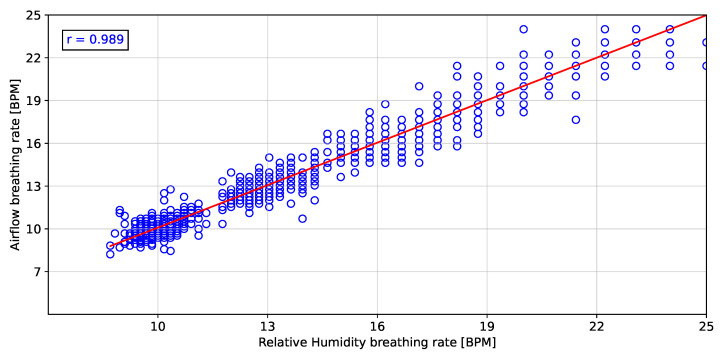
Pearson correlation of the breathing rate from the relative humidity signal respect to the airflow signal.

**Figure 12:**
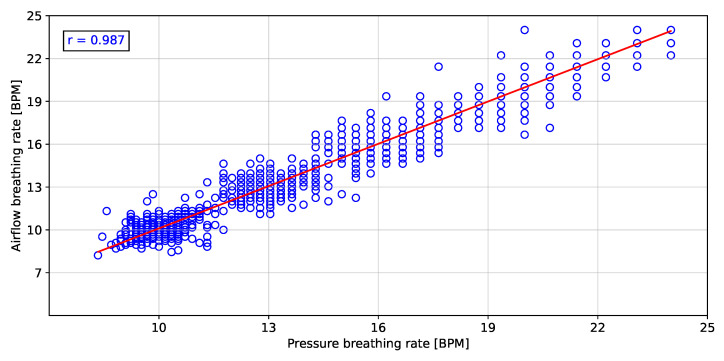
Pearson correlation of the breathing rate from the pressure signal respect to the airflow signal.

**Figure 13:**
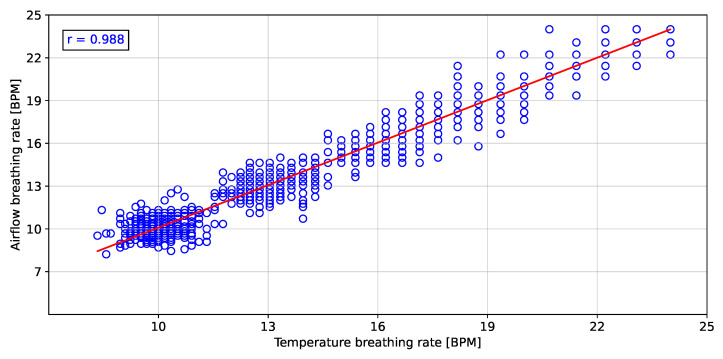
Pearson correlation of the breathing rate from the temperature signal respect to the airflow signal.

To validate the instantaneous breathing rate measurements made with the embedded electronic device, the linear correlation and error analysis were evaluated in comparison to the airflow transducer for the different breathing rates. Figure 11, Figure 12 and Figure 13 show the linear correlation between the instantaneous breathing rates measured for each subject with respect to the instantaneous breathing rates from the reference AF signal. The obtained linear correlation were similar, with 0.989 for the rH signal (see Figure 11), 0.987 for the P signal (see Figure 12) and 0.988 for the T signal (see Figure 13).

**Figure 14:**
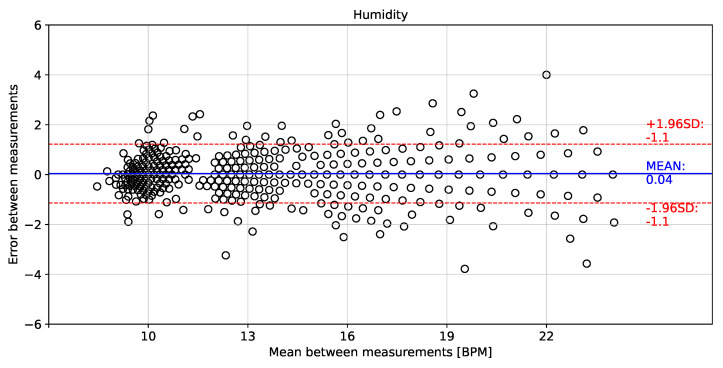
Bland–Altman plot for instantaneous breathing rate using relative humidity signal compared to airflow signal.

**Figure 15:**
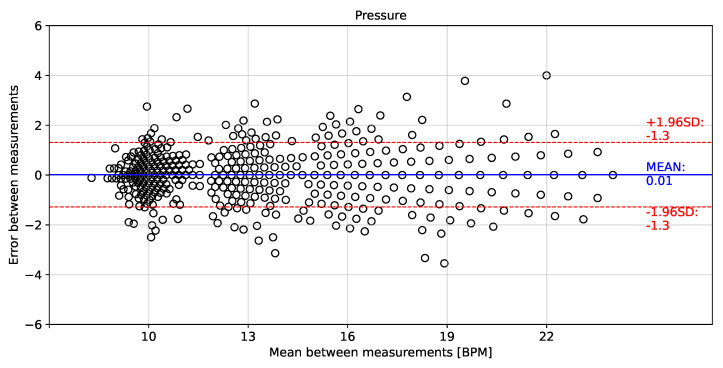
Bland–Altman plot for instantaneous breathing rate using pressure signal compared to airflow signal.

**Figure 16:**
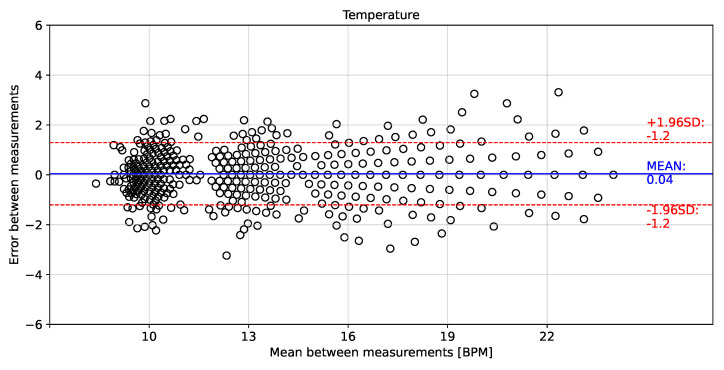
Bland–Altman plot for instantaneous breathing rate using temperature signal compared to airflow signal.

Figure 14, Figure 15 and Figure 16 show the error distributions of the instantaneous breathing rate obtained from each measured signal with respect to the reference AF signal. The rH signal showed a mean error of −0.04 ± 1.1 BPM for calculating the instantaneous breathing signal compared to the AF signal. The P signal showed a mean error of −0.01 ± 1.3 BPM and the T signal showed a mean error of −0.04 ± 1.2 BPM.

### 3.2. Fitting Condition Evaluation

**Figure 17:**
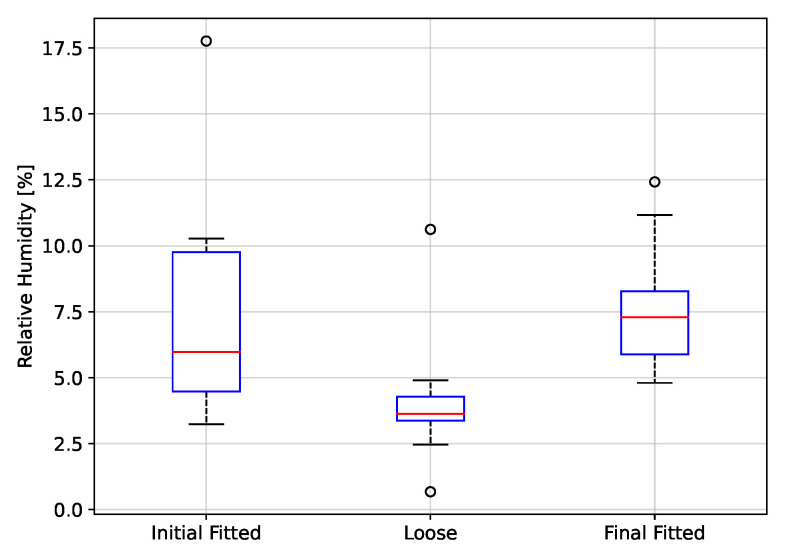
Peak to peak values of relative humidity as a function of fitting condition.

**Figure 18:**
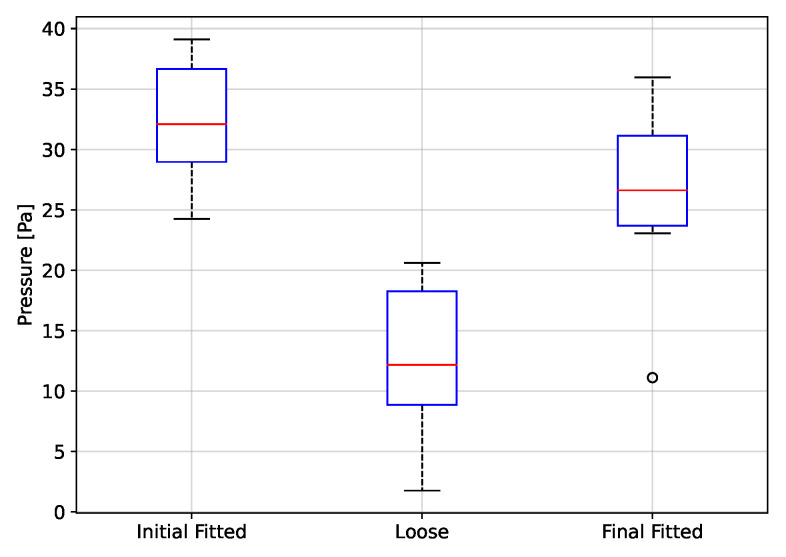
Peak to peak values of pressure as a function of fitting condition.

**Figure 19:**
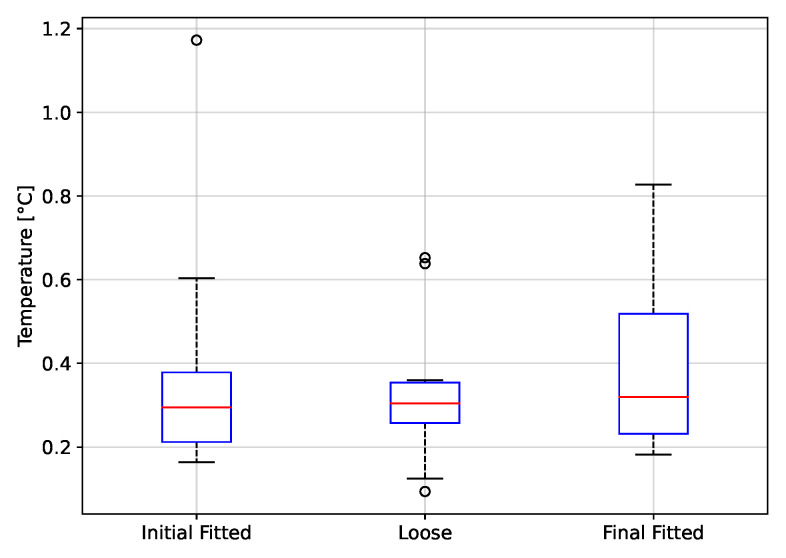
Peak to peak values of temperature as a function of fitting condition.

The peak to peak values of rH, P and T, grouped by fitting condition, are depicted in Figure 17, Figure 18 and Figure 19, respectively. Thus, using rH (Figure 17), the peak-to-peak distribution during the fitted and loose conditions are overlapped, indicating that these two conditions can not be discriminated. The same case, even with more overlap in distribution, occurs using T (Figure 19). On the other hand, using P (Figure 18), there is a clear difference in peak-to-peak distributions between fitted and loose conditions. Even more, the distribution during the initial fitted condition is recovered after the loose condition, when the respirator is fitted again (Final fitted condition).

The t-test results from the fitting condition data groups are shown on Table 1. From the theory from *t*-test, the *p*-values below 0.05 lead to reject the null hypothesis of identical averages for two related conditions (in the columns). Thus, according to p-value in Table 1, the initial fitted condition can be discriminated from the loose condition only using rH and P signals. Moreover, the loose condition can be discriminated from the final fitted condition using rH and P signals. Finally, the P signal is the only variable that permits to see the final fitted distribution is close to initial fitted condition.

In conclusion, the T variable does not achieve any expected results in the fitting condition evaluation, but the P variable permits to evaluate any change of fit condition.

### 3.3. Filter Clogging Evaluation

**Figure 20:**
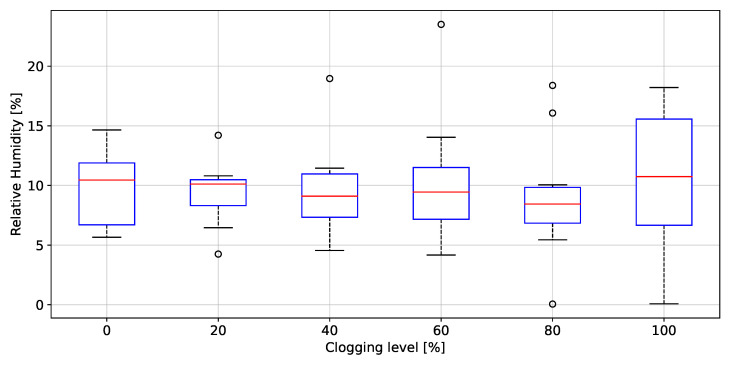
Peak to peak values of relative humidity as a function of the clogging level.

**Figure 21:**
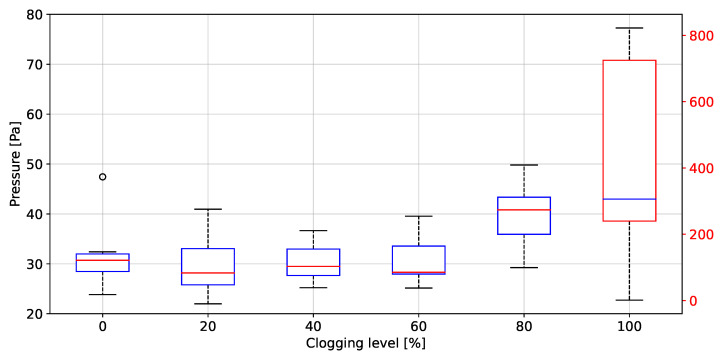
Peak to peak values of pressure as a function of the clogging level.

**Figure 22:**
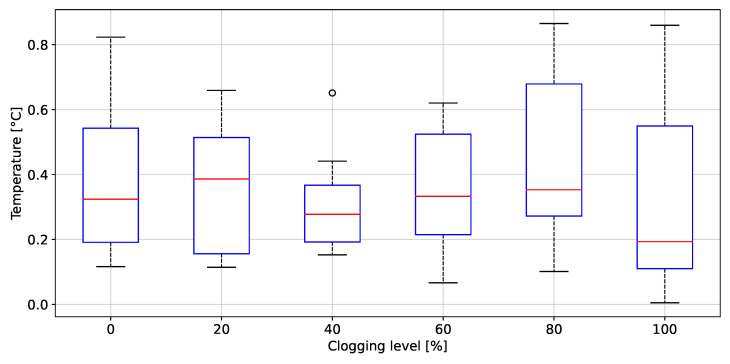
Peak to peak values of temperature as a function of the clogging level.

**Figure 23:**
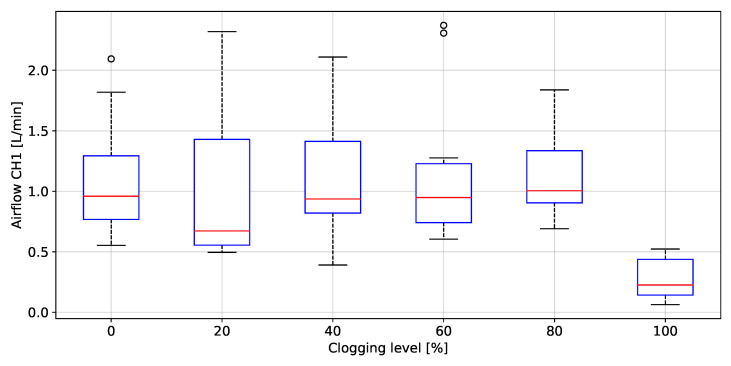
Peak to peak values of airflow (transducer left) as a function of the clogging level.

**Figure 24:**
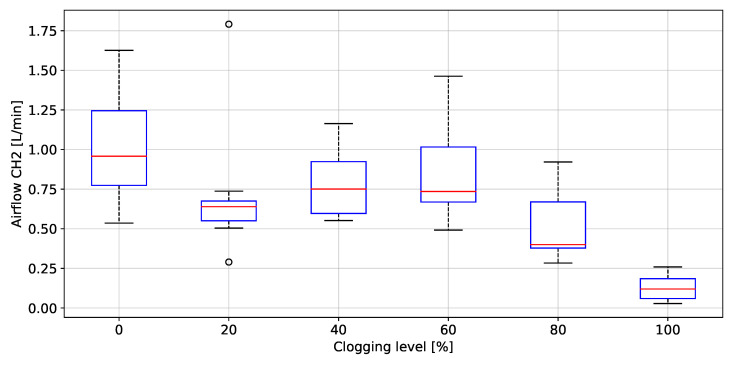
Peak to peak values of airflow (transducer right) as a function of the clogging level.

The peak to peak values of rH, P, T and AF grouped by clogging level, are depicted in Figure 20, Figure 21, Figure 22, Figure 23 and Figure 24, respectively. The ANOVA results are shown on Table 2. The *p*-values (<0.05) showed no difference between clogging levels for the rH and T signals, whereas for the P and A.F. signals there was a significant difference between the groups. The post hoc multiple comparison test (Tukey’s method) for the P signal was performed without including the 100% clogging level, since the distribution for that level it was more than 10 times higher than for the other clogging levels, which hid the existing difference between 0%, 20%, 40%, 60% and 80%, shown in Table 3. The multiple comparison test for the AF signal indicated that the 0%, 20%, 40%, 60% and 80% clogging levels were separable from 100% of clogging level (see Figure 23 and Figure 24) as shown Table 4 and Table 5. Figure 21 shows that the breathing pressure increase 10 times when the filter clogging is close to 100% and the person increase the breathing effort. This feature helps to detect the high filter clogging condition because the people in this condition normally increase their breathing effort unconsciously.

## 4. Discussion

In the present work, a reusable embedded electronic device was designed and implemented for breathing activity monitoring inside an industrial reusable respirator. The proposed system allows measuring the changes in the pressure (P), temperature (T) and relative humidity (rH) in the confined space volume inside a standard industrial respirator, and sending the real-time measurement data wirelessly to an external device for processing and data analytics. Breathing frequency, fitting condition and filter’s clogging level are estimated and reported using the measured data. These data can be used to develop modern plans and strategies with the aim of improving the occupational safety and health throughout the real-time and on-line monitoring of workers’ breathing conditions.

Proposed hardware design considers a battery energy autonomy that exceeds the power requirements for monitoring during a standard working day shift (8 to 12 h [24]). The MCU, pressure/temperature/relative humidity sensors, and the Bluetooth BLE link consume 9.9 mW average during connection and send of data. This allows an autonomy up to 30 h using a 3.7 V-100 mAh battery using a sample frequency of 10 Hz. Furthermore, the hardware was embedded in epoxy resin to homologate the IP66 ingress protection code (protected from total dust ingress, protected from high-pressure water jets from any direction), thus enabling to wear the device in adverse industrial environment conditions in different reusable respirators.

The proposed processing technique is an easy-to-implement method in embedded electronic devices. For measured signals, there is no need for further processing over raw data than the application of a zero-phase 4th Butterworth filter (passband 0.16–0.36 Hz), and a gradient-based algorithm to detect local maxima and minima for T and rH signals, and detection of inflection points for AF and P signals in each breathing cycle. These characteristics make the algorithm suitable for real-time applications. Furthermore, a high linear correlation (0.98) respect to the the reference AF signal was obtained using the three signals (rH, T and P signals) to calculate the breathing frequency (see Figure 11, Figure 12 and Figure 13). Thus, it is possible to accurately calculate the instantaneous breathing rate using any of the signals acquired with the embedded electronic device. The minimum estimation error for breathing activity (0.01 ± 1.3 BPM) was obtained in experimental tests using the P signal as input for the prediction model (see Figure 15). It should be pointed out that the breathing rate calculation can be used for assessing the workers breathing in the workplace during their normal activities and to early-detect possible breathing disorders or deviations in operating conditions.

Using the proposed system, it is possible to discriminate between a fitted and loose condition of the respirator. The test protocol to evaluate the fitting condition considers a first period using a fitted respirator (Initial good fitted condition), then the respirator was loosen and next the respirator was again fitted (Final fitted condition). For the rH and P signals it was possible to separate the Initial Fitted to Loose condition, whereas for the T signal it was not possible to discriminate between fitting conditions. The Initial Fitted to Final Fitted condition t-test was performed to evaluate if the signals peak to peak values returned to their initial distribution when the respirator was adjusted a second time. This was observed only for the P signal. Detecting the fitting condition of a respirator in real-time can help to avoid the exposure of a worker to a harsh environment conditions of dust and gases.

Regarding the clogging level of filters, only using the P signal a statistical difference between the groups 0 to 60%, 80% and 100% clogging level was found. This statistical difference allows to detect three different clogging levels of filters from a reusable industrial respirator. Today, according to the state-of-the-art, there is no device or test or combination of both which can detect clogging levels of filters of an industrial reusable respirator during the work-shift when workers are on duty. The proposed embedded electronic device for breathing activity monitoring can be use to detect the clogging level in real-time and generating early-detection alerts accordingly in cases where the filters are reaching dangerous clogging levels and the workers breathing can be impaired. Laboratory tests have shown that the proposed embedded electronic device could make real-time on-line breathing frequency estimations with good accuracy and precision, discriminates the industrial respirator fitting condition considering two opposite fitting conditions (Fitted and loosened respirator) and identifies of up to three filter clogging levels.

To our knowledge, currently, there are no devices available at the market that allow continuous breathing monitoring in industrial environments, neither to evaluate the respirator and filters performance. Compared to medical respiratory techniques and commercially available equipment, the proposed electronic device allows a continuous and in-site measurement of the worker’s breathing. This continuous monitoring will allow to generate alerts in case of breathing problems and to make plans to reduce breathing diseases. Table 6 shows a performance comparison of breathing rate estimation between our proposal and state-of-art systems. Even though our system has not the best performance to calculate the breathing rate, but 98.9% is high enough for this industrial application. In addition, the embedded electronic device allows for the respirator fitting and filter clogging assessment, which any state-of-art systems do not. While this paper presents promising results, it does have some limitations. The proposed embedded electronic device for workers’ breathing monitoring was only tested with ten healthy subjects breathing in a sit position. Its mandatory to validated with a higher number of subjects in an industrial environment to prove its applicability outside the laboratory environment. Future work may include, and is not limited to, adding new sensors, test new positioning for sensors, and/or integrates inertial sensors for more comprehensive analysis of breathing activity, fitting or clogging of filters of industrial reusable respirators. Additionally, the measured data can be exploited using new data-driven machine learning models to add new features such as better forecast horizon for future clogging levels and prediction of remaining useful life of filters. Another interesting topic for industrial testing and scale-up is the performance evaluation using different wireless communication technologies (WiFi, ZigBee or LoRa instead of Bluetooth to link the device with mobile smartphones or similar). Next steps also include pilot testing at industrial scale in order to validate the system performance, and to adapt the proposed system to other environmental extreme conditions to which workers may be exposed at industrial environments.

## 5. Conclusions

This work proposes a functional building block with embedded electronics capable of measuring and monitoring the breathing activity variables and wear condition of workers when installed in the confined space volume inside an industrial reusable respirator. The design includes pressure, temperature and relative humidity sensors and a Bluetooth BLE communication link to send the collected signals wirelessly to an external device (server cloud, server on-premise or mobile smartphone) for further processing and automated data analytics. After signal pre-processing, instantaneous breathing rate is calculated and respirator fitting and clogging levels of the filter can be estimated. The embedded electronic device was tested with ten healthy participants, presenting promising results about the calculation of breathing rates and the estimation of filters clogging levels and respirator fitting. The results showed that P, T, and rH signals can be used for an accurate rate breathing estimation and detect fitted or loose respirator status. The minimum estimation error for breathing activity (0.01 ± 1.3 BPM) was obtained in experimental tests using the P signal as input for the prediction model. It should be pointed out that the breathing frequency calculation can be used for assessing the workers breathing in the workplace during their normal activities and to early-detect possible breathing disorders or deviations in operating conditions. P was the only signal that allowed to estimate differences between three different filter clogging levels (<60%, 80% and 100% clogging). With the proposed system in this paper we cannot detect filter clogging under 60%. The embedded electronic device is designed to connect with a smartphone to alert workers locally about breathing problems or PPE failure. In addition, this proposed system can alert any issue to supervisors through an internet connection. Then, in case of an internet connection problem, the worker’s supervisor will not be alerted immediately. In industrial sites, the internet connection may fail due to electric noise, network saturation, and areas without internet coverage. For special applications, different wireless communication technologies must be studied and added to the electronic device for a proper work.

Today, according to the state-of-the-art, there is no device or test or combination of both which can detect clogging levels of filters of an industrial reusable respirator during the work-shift when workers are on duty. The proposed embedded electronic device for breathing activity monitoring can be use to detect the high clogging level in real-time and generating early-detection alerts accordingly in cases where the filters are reaching dangerous clogging levels and the workers breathing can be impaired. Those alerts can be sent to the supervisor and the same worker using a mobile phone.

## 6. Patents

Patent Pending, application number 202003196, Instituto Nacional de Propiedad Industrial Chile.

## Figures and Tables

**Table 1:** Two-sided paired t-test performed on fitting condition data groups.

	Initial Fitted to Loose			Loose to Final Fitted			Initial Fitted to Final Fitted		
	**T**	***p*** **-Value**	**df**	**T**	***p*** **-Value**	**df**	**T**	***p*** **-Value**	**df**
Humidity	3.29	0.0094	9	−5.44	0.00041	9	−0.23	0.82	9
Pressure	6.82	$7.72\times 10^{-5}$	9	−5.27	0.00052	9	2.51	0.033	9
Temperature	0.66	0.52	9	−1.42	0.19	9	−0.24	0.81	9

**Table 2:** One-way Analysis of Variance performed on clogging level groups.

	df between Groups	F	*p*-Value
Humidity	5	0.176	0.971
Pressure	4	18.30	$1.385\times 10^{-10}$
Temperature	5	0.47	0.79
Airflow CH1	5	4.38	0.0020
Airflow CH2	5	12.11	$6.92\times 10^{-8}$

**Table 3:** Multiple Comparison Test (Tukey’s method) performed on clogging level groups from Pressure signals.

	0	20	40	60	80
0	1.000000	0.900	0.900000	0.900000	0.005402
20	0.900000	1.000	0.900000	0.900000	0.001000
40	0.900000	0.900	1.000000	0.900000	0.002096
60	0.900000	0.900	0.900000	1.000000	0.002156
80	0.005402	0.001	0.002096	0.002156	1.000000

**Table 4:** Multiple Comparison Test (Tukey’s method) performed on clogging level groups from Airflow CH1 signals.

	0	20	40	60	80	100
0	1.000000	0.900000	0.900000	0.900000	0.90000	0.008029
20	0.900000	1.000000	0.900000	0.900000	0.90000	0.012144
40	0.900000	0.900000	1.000000	0.900000	0.90000	0.011862
60	0.900000	0.900000	0.900000	1.000000	0.90000	0.004099
80	0.900000	0.900000	0.900000	0.900000	1.00000	0.008440
100	0.008029	0.012144	0.011862	0.004099	0.00844	1.000000

**Table 5:** Multiple Comparison Test (Tukey’s method) performed on clogging level groups from Airflow CH2 signals.

	0	20	40	60	80	100
0	1.000000	0.151323	0.416844	0.801411	0.002737	0.001000
20	0.151323	1.000000	0.900000	0.787558	0.641409	0.001000
40	0.416844	0.900000	1.000000	0.900000	0.312879	0.001000
60	0.801411	0.787558	0.900000	1.000000	0.081196	0.001000
80	0.002737	0.641409	0.312879	0.081196	1.000000	0.042081
100	0.001000	0.001000	0.001000	0.001000	0.042081	1.000000

**Table 6:** State-of-the-Art performance comparison of breathing rate estimation.

System	Performance Metric	Value (Best)	Error [BPM]	Reference
Our Proposal	Regression Coefficient	98.9%	0.01 ± 1.3	Biopac Flowmeter
Kundu et al. [9]	% of breathes detected	100%	Non reported	Spirometer
Al-Halhouli et al. [11]	Regression Coefficient	99.22%	0.082 ± 0.109	Nasal e-Health sensor
Massaroni et al. [12]	% of breathes detected	97%	<±3	SpiroQuant system
Harbour et al. [15]	% of breathes detected	99.8%	Non reported	Cosmed Quark system
Hurtado et al. [16]	% of breathes detected	95%	0.4 ± 0.45	Thermistor based
Xu et al. [18]	% of breathes detected	99.7%	0.449 ± 0.620	Self counted breathes

## Data Availability

Not applicable.
